# Benefits, Barriers and Enablers of Breastfeeding: Factor Analysis of Population Perceptions in Western Australia

**DOI:** 10.1371/journal.pone.0088204

**Published:** 2014-02-07

**Authors:** Alison Daly, Christina Mary Pollard, Michael Phillips, Colin William Binns

**Affiliations:** 1 School of Public Health, Curtin University, Perth, Western Australia, Australia; 2 Department of Health, Perth, Western Australia, Australia; 3 Harry Perkins Institute for Medical Research, University of Western Australia, Perth, Western Australia, Australia; Penang Medical College, Malaysia

## Abstract

**Objective:**

The objective of this study was to investigate knowledge and community perceptions of breastfeeding in Western Australia using a factor analysis approach.

**Methods:**

Data were pooled from five Nutrition Monitoring Survey Series which included information on breastfeeding from 4,802 Western Australian adults aged 18–64 years. Tetrachoric factor analysis was conducted for data reduction and significant associations identified using logistic, ordinal and poisson regression analyses.

**Results:**

Four factors were derived for benefits (it’s natural, good nutrition, good for the baby, and convenience), barriers (breastfeeding problems, poor community acceptability, having to go back to work, and inconvenience) and for enablers (breastfeeding education, community support, family support and not having to work). As assessed by standardized odds ratios the most important covariates across benefit factors were: importance of breastfeeding (ORs range from 1.22–1.44), female gender (ORs range from 0.80 to 1.46), being able to give a time for how long a baby should be breastfed (ORs range from 0.96 to 1.27) and education (less than high school to university completion) (ORs range from 0.95 to 1.23); the most important covariate across barrier factors was being able to give a time for how long a baby should be breastfed (ORs range from 0.89 to 1.93); and the most important covariates across all enabling factors were education (ORs range from 1.14 to 1.32) and being able to give a time for how long a baby should be breastfed (ORs range from 1.17 to 1.42).

**Conclusions:**

Being female, rating breastfeeding as important, believing that babies should be breastfed for a period of time and education accounted for most of the statistically significant associations. The differences between male and female perceptions require investigation particularly in relation to returning to work.

## Introduction

The promotion of breastfeeding is an international public health priority and the recommendation to exclusively breastfeed until around six months of age has been adopted by many countries around the world including Australia [1], [2]. The recommendation regarding the length of time to continue breastfeeding after the introduction of solid foods varies, for example, until twelve months of age and beyond in Australia [1] and the United States [3], and to continue breastfeeding to the age of two years or beyond which is the World Health Organization (WHO) recommendation that has been adopted by many developing countries as well as countries like Canada [2].

The strength of evidence to support the promotion of breastfeeding is growing and compelling, particularly as breastfeeding benefits both the baby and the mother. Apart from breast milk being the ideal food for optimal infant growth and development [4], there are additional long-term benefits for the infant. There is convincing evidence of a lower risk of becoming obese [5] or developing high cholesterol or high blood pressure [6] later in life. Breastfeeding is also associated with lower rates of mortality and morbidity from gastrointestinal infections for the baby [7], [8] and reduced risk of coeliac disease [9] and asthma [10], [11]. There is some evidence that breastfed babies have improved cognitive development [12], [13], and increased bonding with the mother [14]. Benefits for the mother include a reduced risk of ovarian cancer, quicker recovery after birth, and a possible reduced risk of breast cancer and type II diabetes [1]. There is also evidence that breastfeeding is associated with a lower risk of Sudden Infant Death syndrome [10]. Evidence to date shows no counter-indications for exclusive breastfeeding for around six months for healthy full-term babies [15], [16].

Population based surveys are able to provide specific information about areas of interest within a community. They can identify population groups considered to be at health risk due to their behaviours [17]. Although questions on breastfeeding have been included in population surveys before, respondents are generally women of child bearing age or with small babies. The topic seems to be considered less relevant to the general population [18], [19]. Surveys rarely ask the public about the perceived benefits of breastfeeding or circumstances that make it easier or more difficult to breastfeed. If the general public do not know the benefits of breastfeeding, messages about the importance of breastfeeding are likely to be less compelling and effective in facilitating exclusive breastfeeding for the recommended six months. Without knowledge of the potential benefits and barriers, complying with the breastfeeding guidelines may be difficult for mothers.

The Health Department of Western Australia conducts triennial population surveys of men and women aged 18 to 64 years to guide the development of interventions to increase behaviours consistent with the Australian Dietary Guidelines (Nutrition Monitoring Survey Series-NMSS). These unique surveys explore knowledge about breastfeeding recommendations, barriers and enablers of breastfeeding from women currently breastfeeding, potential mothers, their partners and the population past the child-bearing age.

The objective of this study was to investigate the perceptions of breastfeeding in the general community of Western Australia (WA) using a factor analysis approach. We were particularly interested in assessing perceptions of factors which may encourage or deter women from breastfeeding.

### Ethics Statement

The NMSS were granted approval from the Western Australia Department of Health Human Research Ethics Committee (HREC) who act in accordance with the National Health and Medical Research Council (NHMRC) Ethics Committee guidelines. As part of that NHMRC ethics procedure, consent issues are addressed and specifically, our procedure for receiving verbal consent from participants was approved.

## Methods

### Study Population

Five cross-sectional computer assisted telephone surveys were conducted with over 1200 WA adults aged between 18 and 64 years during July and August in the years 1995, 1998, 2001, 2004 and 2009. A total of 5496 people were surveyed in this pooled Nutrition Monitoring Survey Series (NMSS) of which 4208 provided information on all of the variables used in the inferential analysis. All of the variables had missing values less than 1% except income (8%) and the rating of importance of breastfeeding (3%). Using computer generated random digit dialling with known area prefixes, the 1995, 1998 and 2001 samples were stratified by area and the 1998 and 2001 samples were also quota sampled by sex. Using the most recently available Electronic White Pages, the 2004 and 2009 samples were randomly selected by area and the 2004 survey quota sampled by area and sex. In 2004 and 2009 all sample households with an address were sent an approach letter explaining the purpose of the survey, how the sample was selected and how long the interview would take. In 2004 eligible respondents within a household were selected by the most recent birthday and no substitutes were accepted unless the quota had been achieved for that group. In 2009 eligible respondents within a household were selected by the most recent birthday and no substitutes were accepted. There were no partially completed interviews. The response rate ranged from 29.5% (1998) to 87.8% (2009) with an average of 50.4%.

### Measures

The NMSS monitors population attitudes, beliefs and selected self-reported behaviours. In relation to this study the questionnaire contains questions about breastfeeding including a rating of the importance of breastfeeding and an opinion of how long a baby should be breastfed. Three multiple-response questions were asked about benefits, barriers and enablers of breastfeeding:

1. What do you think are the benefits of breastfeeding for babies?
2. What do you think makes it difficult for women to continue to breastfeed their babies for at least six months? (barriers)
3. What do you think would make it easier for women to continue to breastfeed their babies for at least six months? (enablers)

The data collection evolved over time. The initial survey questionnaire in 1995 contained open-ended questions which asked each respondent to identify as many benefits, barriers and enablers in relation to breastfeeding as they could. Interviewers were instructed to probe for as many responses as possible. The multiple responses were grouped into categories assigned by the researchers and dietitians based on focus group research conducted in Perth, Western Australia which identified perceptions of barriers and promoters at the time [20], [21]. For each question a number of categories were identified. Since 1995, the same question format has been used with interviewers pre-coding responses into these identified categories. Interviewers were instructed to record verbatim any responses that didn’t fit into the categories. These ‘other’ responses were then recoded into the existing categories where possible by an expert panel. There was an average of 3.9% on each occasion that were unable to be recoded and remained as an ‘other’ category. The ‘other’ category is not included in the analysis.

For the purpose of this study, we interpret ‘knowledge of breastfeeding’ as knowing something about the benefits, barriers and enablers as well as rating breastfeeding as important and having an opinion that babies should be breastfed for a specific time.

### Analysis

Due to the complex sampling designs the data were weighted using adjustments for differing sampling fractions for areas of residence (all years) and for probability of selection of the household from the number of listings in the electronic White Pages and the number of adults (ages 18–64) within the household (2009 only). Post-estimation adjustment was used to correct for under or over representation of gender, age and areas of residence using the 2011 Estimated Resident Population for WA aged 18–64 years (the year of the most recent census at the time of analysis) [22].

The plan for the analyses specified a four stage approach as follows: First we examined individual knowledge, barriers and enablers by gender; secondly, to reduce the data, tetrachoric factor analysis was conducted to identify groupings within knowledge, barriers and enablers; thirdly ordinal regression was used to examine each of the factors for statistically significant sociodemographic associations; finally the total number of responses to knowledge, barriers and enablers were examined to see if the number mentioned was statistically significantly associated with any of the sociodemographic indicators and to see whether the number of each increased or decreased over time.

Descriptive statistics used estimates of prevalence with 95% confidence intervals. Logistic, poisson and ordinal regression analyses were conducted using the methods which correct for sample design and post survey weighting. Pearson chi squared tests were used to estimate *p* values and to determine statistical significance in the univariate tables.

Logistic regression was used to investigate single benefits, barriers or enablers where there were statistically significant differences between males and females. As the benefits, difficulties and enablers were all multiple response variables and recorded as 0 = No, 1 = Yes, a tetrachoric factor analysis using varimax rotation was conducted to reduce the data and identify any underlying factors [23]. Ordinal logistic regression analyses were conducted on the factors extracted because the factor scores were based on the sum of the questions within each factor making an ordinal assumption for the scale more conservative than an assumption of an interval scale [24]. Each of the factors was entered into ordinal logistic regression analysis to identify the variables associated with each factor score. The socio-demographic variables entered into the model were gender (male compared with female), age in groups (18–24, and 25–64 in five year groups), highest level of education attained (four groups from less than year 10 schooling to a completed university degree), household income (earning less than Aus$60,000 per annum compared with earning Aus$60,000 or more), employment status (in paid employment compared with not currently in paid employment), country of birth (Australia compared with all other countries of birth) and area of residence (metropolitan Perth compared with outside that area). Two other variables were also included, rating of the importance of breastfeeding (1 = not at all important to 5 = very important) and not knowing how long a baby should be breastfed compared with being able to give a specific time for how long a baby should be breastfed. Dichotomous variables are coded with first category = 0 and the second category = 1. The validity of the proportional odds assumption for ordinal logistic regression was tested using the adjusted Wald statistic and the assumption of linearity was tested for education using fractional polynomial transformations. Standardized odds ratios are reported to enable the relative importance of the independent variables to be assessed. To avoid inflating the overall critical p value, multiple comparisons were corrected using the method of Holm [25].

In the results section only those *p* values which were significant after correction are reported. Heckman selection models were used to examine the sensitivity of the results to missing values [26]. After testing for the validity of the assumption of a Poisson distribution, poisson regression analysis was conducted to identify predictors of the total number of benefits, barriers and enablers.

A p value less than 0.05 was regarded as statistically significant. All analysis was conducted using the Stata statistical package (Version 12, StataCorp LP, College Station, Tx).

## Results

Table 1 describes the NMSS survey sample characteristics across the pooled dataset from 1995 to 2009.

**Table 1:** Sample Characteristics by Socio-Demographic Groups, NMSS 1995–2009.

	Sample	%
**Gender**	**5496**	
Male	2430	44.2
Female	3066	55.8
**Age group**	**5496**	
18–24 years	521	9.5
25–34 years	1124	20.5
35–44 years	1565	28.5
45–54 years	1306	23.8
55–64 years	980	17.8
**Highest level of education**	**5472**	
Less than Year 12	1546	28.3
Year 12 or equivalent	1188	21.7
Trade/Certificate/Diploma	940	17.2
University	1798	32.9
**Household income**	**5054**	
Up to $60,000	2861	56.6
Over $60,000	2193	43.4
**Employment status**	**5491**	
Employed	3973	72.4
Unemployed	1518	27.6
**Country of birth**	**5495**	
Born in Australia	3724	67.8
Born elsewhere	1771	32.2

**Figure 1:**
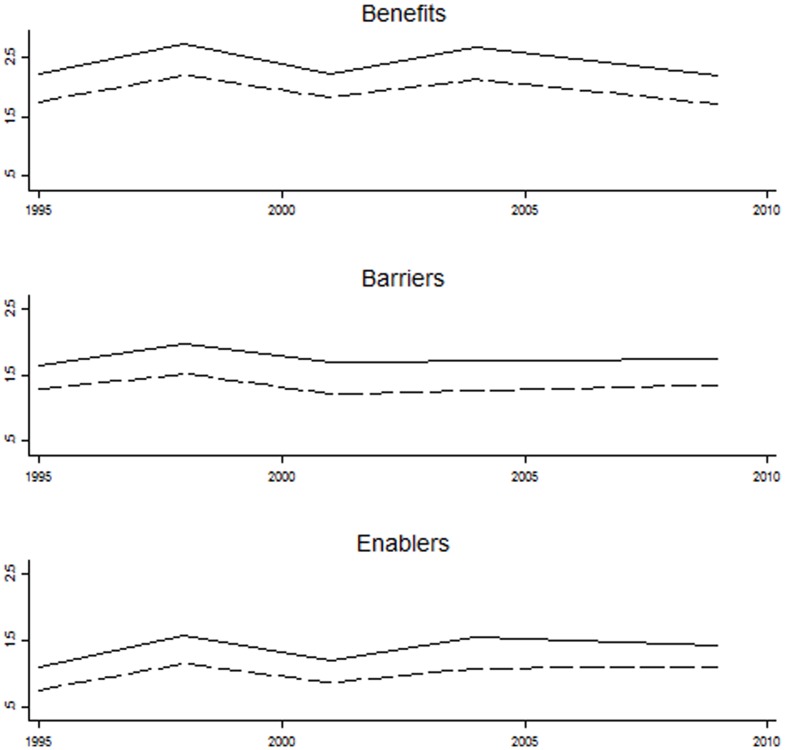
Mean number of benefits, barriers and enablers by gender and year, NMSS 1995–2009. Y axis : Mean number. X axis: Year of survey. Legend: Solid line = Females; Dashed line = Males.

Although there were changes in the proportion of people choosing each benefit, barrier and enabler in different years there were no consistent linear trends over time for either males or females (Figure 1). Nevertheless the year of survey (1995, 1998, 2001, 2004 and 2009) was included in the inferential analyses to adjust for any small variation over time in the pooled dataset.

Table 2 shows the proportion of men and women choosing each benefit, barrier and enabler with the confidence interval around each estimate.

**Table 2:** Benefits, barriers and enablers of breastfeeding by gender, NMSS 1995–2009^a^.

	Male	Female
**Benefits for baby of breastfeeding**	**(%)CI**	**(%)CI**
Provides Immunity	37.8 (35.7–40.0)	60.8 (58.7–62.9)
Provides vitamins and minerals	39.9 (37.7– 42.2)	41.0 (38.9–43.2)
Ideal Food	23.2 (21.3–25.2)	25.3 (23.5–27.3)
Good for baby’s health	29.6 (27.5–31.7)	34.8 (32.7–36.9)
Natural/No chemicals	22.3 (20.5–24.3)	17.5 (15.8–19.3)
Easy/Convenient	5.9 (5.0–7.1)	14.8 (13.2–16.4)
Encourages emotional bonding	34.5 (32.3–36.7)	45.0 (42.8–47.2)
Other	2.3 (1.7–3.1)	5.0 (4.1–6.0)
**Barriers to breastfeeding**		
Need to work	27.2 (24.9–29.6)	48.8 (46.4–51.2)
Problems with milk supply	18.0 (16.1–20.2)	25.7 (23.6–27.8)
Soreness	26.8 (24.5–29.2)	30.8 (28.6–33.2)
Inconvenient	11.1 (9.2–12.3)	10.6 (9.2–12.3)
Not publicly acceptable	23.8 (21.6–26.2)	22.0 (20.1–24.1)
Not enough time	16.0 (14.2–18.1)	21.9 (20.0–24.0)
Don’t like doing it or seeing it	1.5 (.92–2.3)	2.0 (1.4–2.8)
Other	8.2 (6.8–9.8)	13.2 (11.6–15.0)
**Enablers of breastfeeding**		
Not having to work	18.5 (16.8–20.3)	28.1 (26.2–30.1)
Having more time	9.8 (8.5–11.2)	14.8 (13.3–16.5)
Having more facilities	9.5 (8.2–10.9)	13.7 (12.2–15.3)
Having more education	11.9 (10.5–13.4)	18.4 (16.7–20.2)
Being better informed about theprocess	7.4 (6.3–8.7)	9.8 (8.6–11.2)
Having support of partner and family	7.1 (6.0–8.3)	12.8 (11.4–14.4)
Being acceptable to community	28.0 (26.0–30.2)	33.3 (31.2–35.4)
Other	3.6 (2.8–4.5)	5.2 (4.3–6.3)

a Multiple responses allowed.

### Benefits of Breastfeeding for the Baby

A higher percentage of females knew three or more benefits compared with males (48.8% and 32.2% respectively, χ^2^ = 110.1, p<.0001). One third of respondents (33.1% [95% CI 31.6%–34.6%]) knew at least two benefits of breastfeeding while 6.5% [95% CI 5.7%–7.3%] did not know any benefits. A logistic regression analysis found that males (OR 3.7 p<.0001), people aged between 18 and 34 years (OR 1.96 p<.0001), people having only school education (OR 1.88 p<.0001) and those surveyed in 2001 (OR 1.64 p<.05) or 2009 (OR 2.5 p<.0001) were more likely to have no knowledge of the benefits of breastfeeding. About the same proportion of males and females and women said breastfeeding provides vitamins and minerals, or is the ideal food for babies. A significantly higher proportion of women than males reported that breastfeeding provides immunity, is easy or convenient, and encourages emotional bonding. Males were more likely than females to report that breastfeeding was natural or had no chemicals.

### Barriers

Significantly more women said that the need to work was a breastfeeding difficulty (48.8%) compared to 27.2% of men. Women were also significantly more likely than males to report breastfeeding problems such as problems with milk supply and lack of time, as barriers to breastfeeding. About the same proportion of men and women reported inconvenience, poor public acceptability, and not having enough time as barriers to breastfeeding.

### Enablers of Breastfeeding

Similar patterns were seen with breastfeeding enablers although having breastfeeding more accepted in the community was most often reported by both women and men (33.3% and 28% respectively) as an enabler to breastfeeding, followed by help with breastfeeding problems such as soreness and supply, work and support issues. A logistic regression analysis showed that being female (OR 1.3 p<.001), having a university education (OR 1.6 p<.001), being born outside Australia (OR 1.3 p<.001) and being surveyed after 1995 (OR 1.04 p<.001) were all associated with believing that greater community acceptance would make breastfeeding easier.

### Underlying Factors Influencing Breastfeeding

The tetrachoric correlation based factor analyses identified four factors each for benefits, barriers and enablers to breastfeeding. Table 3 shows the four factors associated with them and the Eigen value and the explained variance for each.

**Table 3:** Factors which underlie the benefits, barriers and enablers of breastfeeding, NMSS 1995–2009.

Benefits for baby of breastfeeding	Factor one	Factor two	Factor three	Factor four
Factor Name	Natural	Nutrients & bonding	Good for baby	Convenient
Category(ies)	Natural	Vitamins/minerals & bonding	Good for baby’shealth & ideal food	Easy & convenient
Eigen value	1.3	1.7	1.1	0.94
Variance Explained (total 0.934)	0.38	0.27	0.19	0.1
**Barriers to breastfeeding**	**Factor one**	**Factor two**	**Factor three**	**Factor four**
Factor Name	Breastfeeding problems	Unacceptable	Work	Inconvenience
Category(ies)	Supply problems and breast soreness	Dislike breastfeeding & unacceptable	Have to work	No time and breastfeeding inconvenient
Eigen value	1.5	1.3	1.1	0.97
Variance Explained (total 0.960)	0.42	0.28	0.19	0.07
**Enablers of breastfeeding**	**Factor one**	**Factor two**	**Factor three**	**Factor four**
Factor Name	Education	Community support	Family support	Not having to work
Category(ies)	More education about breastfeeding generally	More facilities &public acceptance	Having more time & family support	Not having to work
Eigen value	1.7	1.44	1.16	0.82
Variance Explained	0.39	0.29	0.19	0.07

### Variables Associated with the Benefit Factors of Breastfeeding

Benefit factor one relates to the naturalness of breastfeeding and the fact that breast milk is free from chemicals. There is a significant association between the factor score and decreasing year of survey from 2009 (OR = 0.853 *p* = 0.013), being male (Reciprocal OR = 1.25 *p*<0.013), having an income greater than $60,000 (OR = 1.18 *p* = 0.007) and increasing rating of the importance of breastfeeding (OR = 1.29 *p*<0.001). Benefit factor two relates to breast milk providing nutrients for the baby and emotional bonding with the mother. There is a significant association between the factor two score and decreasing year of survey from 2009 (OR = 0.857 *p* = 0.002), being female (OR = 1.09 *p* = 0.042), increasing education level (OR = 1.22 *p*<0.001), increasing rating of the importance of breastfeeding (OR = 1.35 *p<*0.001*)* and being able to give a specific time for how long a baby should be breastfed (OR = 1.19 *p*<0.001). Benefit factor three relates to the health effects of breastfeeding for the baby and that breast milk is an ideal food. There is a significant association between the factor score and being female (OR = 1.46 *p*<0.001), increasing age in five year increments (OR = 1.17 *p* = 0.001), increasing education level (OR = 1.23 *p*<0.001), increasing rating of the importance of breastfeeding (OR = 1.44 *p*<0.001) and being able to give a specific time for how long a baby should be breastfed (OR = 1.27 *p*<0.001). Factor four relates to the ease and convenience of breastfeeding. There is a significant association between the factor four score with being female (OR = 1.18 *p*<0.001), increasing level of education (OR = 1.11 *p* = 0.024), increasing rating of the importance of breastfeeding (OR = 1.22 *p*<0.001) and being able to give a specific time for how long a baby should be breastfed (OR = 1.20 *p* = 0.001). As assessed by standardized odds ratios the most important covariates, across all benefit factors were: the importance of breastfeeding (ORs range from 1.22–1.44), female gender (ORs range from 0.80 to 1.46), being able to give a specific time for how long a baby should be breastfed (ORs range from 0.96 to 1.27), and increasing education level (less than high school to university completion) (ORs range from 0.95 to 1.23). Employment status, country of birth and area of residence were not associated with any breastfeeding benefit factors.

### Variables Associated with the Barrier Factors for Breastfeeding

Barrier factor one relates to milk supply and breast soreness. There is a significant association between the factor one score and being able to give a specific time for how long a baby should be breastfed (OR = 1.13 *p*<0.001). Barrier factor two relates to breastfeeding being distasteful and unaccepted by society. There is no significant association between the factor two score and any of the independent variables after correction for multiple comparisons. Barrier factor three relates to needing to work. There is a significant association between the factor three score and being female (OR = 1.60 *p*<0.001), increasing age (OR = 1.26 *p* = 0.002), increasing education (OR = 1.36 *p*<0.001), and being able to give a specific time for how long a baby should be breastfed (OR = 1.16 *p* = 0.021). Barrier factor four relates to the inconvenience of breastfeeding. There is a significant association between this factor and being able to give a specific time for how long a baby should be breastfed (OR = 1.93 *p* = 0.002). As assessed by standardized odds ratios the most important covariate across all barrier factors was being able to give a specific time for how long a baby should be breastfed (ORs range from 0.89 to 1.93). There were no associations with year, employment status, household income, country of birth, area of residence and importance of breastfeeding.

### Variables Associated with the Enabling Factors for Breastfeeding

Enabling factor one relates to the necessity of breastfeeding information and education. There is a significant association between this factor and increasing education level (OR = 1.17 *p* = 0.003), increasing rating of breastfeeding importance (OR = 1.28 *p*<0.001) and being able to give a specific time for how long a baby should be breastfed (OR = 1.26 *p*<0.001). Enabling factor two relates to community facilities and community acceptance of breastfeeding. There is a significant association between this factor and increasing levels of education (OR = 1.21 *p*<0.001), increasing rating of breastfeeding importance (OR = 1.24 *p*<0.001) and being able to give a specific time for how long a baby should be breastfed (OR = 1.18 *p*<0.001). Enabling factor three relates to family support and having time to breastfeed. There is a significant association with this factor and being female (OR = 1.25 *p*<0.001), increasing level of education (OR = 1.14 *p* = 0.009), and being able to give a time for how long a baby should be breastfed (OR = 1.42 *p*<0.001). Enabling factor four relates to not having to work. There is a significant association with factor four and increasing year of survey (OR = 1.19 *p* = 0.003), being female (OR = 1.29 *p*<0.001), increasing age (OR = 1.27 *p*<0.001), increasing level of education (OR = 1.32 *p*<0.001), and being able to give a specific time for how long a baby should be breastfed (OR = 1.17 *p* = 0.003). As assessed by standardized odds ratios the most important covariates across all enabling factors were: education (ORs range from 1.14 to 1.32) and being able to give a specific time for how long a baby should be breastfed (ORs range from 1.17 to 1.42). There were no associations with employment status, household income, country of birth and area of residence.

### Changes Over Time

The ordinal regression models showed that survey year was associated with two of the reported benefit factors: factor one relating to the naturalness of breastfeeding and that breast milk is free from chemicals and factor two relating to the provision of nutrients for the baby and emotional bonding with the mother. In both cases there was a decreasing association of these factors with the year of survey. One enabling factor, factor four relating to not having to work, is also related to the year of the survey with an increasing association over time. No other associations between other factors and year of the survey were found.

### Variables Associated with the Total Number of Benefits, Barriers and Enablers

In a multivariate poisson regression analysis of the total numbers of benefits, barriers and enablers (Table 4) the total number of benefits of breastfeeding reported increased with being female, having a university education, and rating breastfeeding as very important. The total number of barriers to breastfeeding increased with the year of the survey, being female, having a university education, living in the metropolitan area and thinking that a baby should be breastfed at least for some time. The total number of enablers to breastfeeding increased with being female, having a university education, being Australian born, living in the metropolitan area, rating breastfeeding as very important and thinking that a baby should be breastfed at least for some time.

**Table 4:** Number of breastfeeding benefits, barriers and enablers mentioned, NMSS 1995–2009.

Total number of benefits mentioned	Coeff.	95% Confidence Interval		*p* value
Year of survey	0.01	−0.02	0.05	0.475
Age in five year groups	0.01	0.00	0.02	0.243
Female versus (vs.) male	0.14	0.11	0.18	0.004
University Education vs. less education	0.13	0.09	0.16	0.017
Income $60,000 or more v. income less than $60,000	0.04	−0.01	0.08	0.272
Born in Australia vs. born overseas	0.03	0.00	0.07	0.146
Living outside metropolitan area vs. metropolitan	−0.03	−0.07	0.01	0.530
Breastfeeding a baby very important vs. less than very important	0.28	0.23	0.34	<0.001
baby should be breastfed for specific time vs. not giving a time	0.29	−0.20	0.36	0.127
Constant	0.12	0.03	0.27	0.037
**Total number of barriers mentioned**				
Year of survey	0.09	0.01	0.18	0.038
Age in five year groups	0.00	−0.02	0.02	0.742
Female vs. male	0.19	0.11	0.28	<0.001
University Education vs. less education	0.19	0.10	0.27	<0.001
Income $60,000 or more v. income less than $60,000	0.01	−0.08	0.10	0.866
Born in Australia vs. born overseas	0.04	−0.04	0.13	0.346
Living in the metropolitan area vs outside	−0.12	−0.22	−0.03	0.011
Breastfeeding a baby very important vs. less than very important	0.08	−0.03	0.19	0.146
baby should be breastfed for specific time vs. not giving a time	0.41	0.23	0.60	<0.001
Constant	−0.24	−0.56	0.09	0.159
**Total number of enablers mentioned**				
Year of survey	−0.02	−0.09	0.05	0.555
Age in five year groups	0.01	−0.01	0.03	0.229
Female vs. male	0.27	0.21	0.34	<0.001
University Education vs. less education	0.25	0.19	0.32	<0.001
Income $60,000 or more v. income less than $60,000	0.04	−0.03	0.11	0.236
Born in Australia vs. born overseas	0.10	0.03	0.17	0.004
Living in the metropolitan area vs outside	−0.11	−0.18	−0.04	0.002
Breastfeeding a baby very important vs. less than very important	0.28	0.18	0.38	<0.001
baby should be breastfed for specific time vs. not giving a time	0.46	0.31	0.61	<0.001
Constant	−0.78	−1.04	−0.52	<0.001

**Figure 2:**
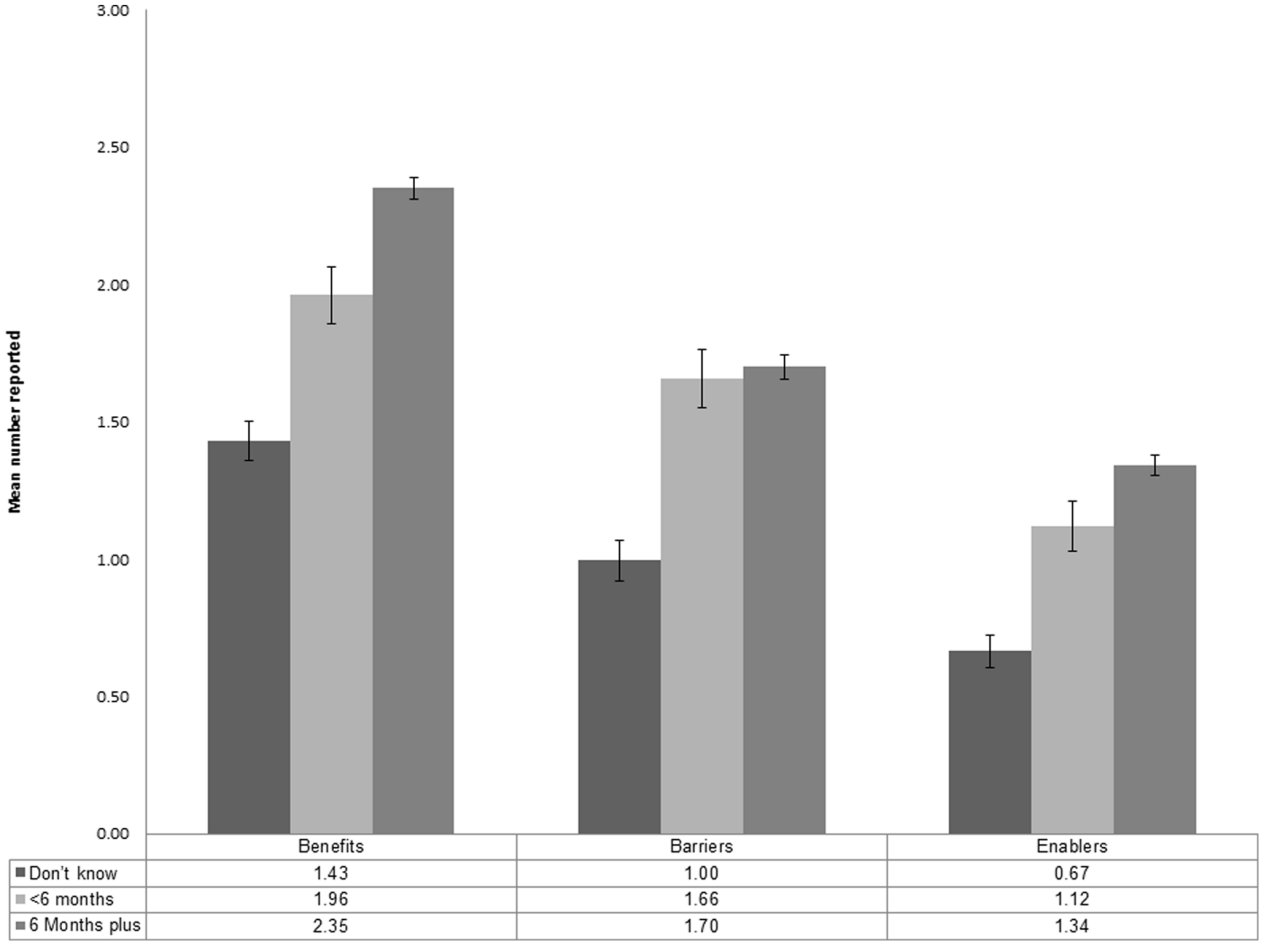
Mean number of benefits, barriers and enablers by how long a baby should be breastfed, NMSS 1995–2009.

Aside from gender and education, two of the most important variables related to the total number of benefits, barriers and enablers that a respondent mentions are the rating of the importance of breastfeeding and the time given that a baby should be breastfed for (duration). The mean number of benefits mentioned by respondents who rated breastfeeding as very important is 2.39 (CI: 2.35–2.42) compared with those who rated it as less than very important 1.69 (CI: 1.62–1.77). The mean number of benefits and enablers increased with increasing time for how long a baby should be breastfed. There was no significant association between time for how a long baby should be breastfed and the mean number of barriers identified (Figure 2).

All regression models were checked for goodness of fit and were satisfactory with *p* values >0.05. The Heckman selection models showed that the results were not sensitive to missing values with none of the Mill’s ratio *p* values <0.05.

## Discussion

The objective of this study was to investigate the perceptions of breastfeeding in the general community of WA using a factor analysis approach in order to assess the relationships between these perceptions and knowledge about breastfeeding. We defined knowledge of breastfeeding as knowing something about the benefits, barriers and enablers as well as rating breastfeeding as important and having an opinion that babies should be breastfed for a specific time.

Our results suggest that the knowledge of the benefits of breastfeeding among the general community was lower than would have been predicted from respondents’ ratings of the importance of breastfeeding. The mean number of benefits reported was less than three (2.39). While believing that a baby should be breastfed for over six months increased the mean number of benefits mentioned, one in fifteen people were not able to mention any benefits of breastfeeding and a further twenty percent only mentioned one benefit. This was in spite of respondents being encouraged to think about as many breastfeeding benefits as possible which leeds to the conclusion that the level of knowledge regarding breastfeeding among the WA population is not high. Females were able to report more benefits than males but less than half could name more than two benefits of breastfeeding. This underestimation of the benefits of breastfeeding has also been reported in Canada [27]. These findings support the need for ongoing community wide education regarding the benefits of breastfeeding to infants and mothers as well as support for comprehensive pre-natal education [28].

The same pattern is shown regarding barriers to breastfeeding. More barriers were reported by women but the mean number of barriers women identified was less than two. This result is somewhat surprising given that in WA, less than fifteen percent (14.8%) of mothers reported exclusively breastfeeding to six months in 2010 and less than half (43.7%) breastfed at all after six months [29]. While the perception of the community may be that there are relatively few barriers to breastfeeding, our results and the low compliance with the Infant Feeding Guideline recommendations to exclusively breastfeed until about six months suggest that they are a major determinant of breastfeeding practice. The main barrier to the continuation of breastfeeding for more than six months was the need to return to work. These findings support previous research showing that even in countries where there is support for maternity leave [30] and here in Australia where it was the second most commonly given reason for stopping breastfeeding [31]. While some Australian mothers report being able to breastfeed and work [29] our results suggest that there is a perception among the community that either mothers would not be supported to continue breastfeeding by their employing organization or would not be able to breastfeed is similar to that found in other studies [31], [32]. The perceived barriers of poor social acceptability, lack of time and needing to return to work may be amenable to change however a comprehensive range of intersectoral interventions, including health system level to support health professionals who support mothers would be required [33]–[35].

For mothers themselves, our results suggest support from family and partners would be beneficial. This is consistent with previous research in Australia [36]. Government policies supporting family-based parental leave, including paternity leave, may help to assist mothers of new born babies address the difficulty of breastfeeding when there were other young children in the family as well as encourage emotional connection with the infant. Australians have access to a 52 week job-protected family leave, and more recently a paid parental leave scheme which enables eligible working parents up to 18 weeks paid minimum wage parental leave or two weeks ‘dad and partner pay’ [37]. A comparison of fathers’ patterns of statutory paternity leave taking across 24 countries between 2003 and 2007 found that taking leave was more likely with at least 50% of income replacement and of greater than 14 days allowance [38].

The current study findings also supports the need for policies to assist the acceptability and feasibility of breastfeeding at work including employer provision of facilities and breaks for women to breastfeed when feasible and practical [39]. Education campaigns regarding the benefits of breastfeeding may also assist as support for such policies is likely to be based on knowledge of the benefits of breastfeeding [2], [39]. Health workers are well placed to assist mothers and families to address the breastfeeding problems. The NHMRC *Infant Feeding Guidelines for Health Workers* acknowledges that they can provide invaluable factual information and empathetic support, demonstrate practical skills and discuss strategies for problem solving [1]. It is important that health workers are trained and encouraged to enable this to happen.

While these results are specific to Western Australia, the findings are consistent with the breastfeeding literature and make them likely to be applicable to women in countries with a similar demographic structure.

The data in this study are cross-sectional and all results in this survey relate to associations rather than causality. Cross-sectional surveys such as the NMSS are consistent with the World Health Assembly resolution to monitor non-communicable diseases and their determinants, and strengthen surveillance systems to provide the foundation for advocacy and policy development, as well as providing a tool to evaluate the effectiveness of interventions and progress made [40].

The main limitation of this study was that the data collection method changed over time and with it the response rates. The lower response rates for years prior to 2009 were mainly due to the Random Digit Dialing method which, particularly for the earlier years, was done without any matching to existing known operational numbers.

The quota sampling in years prior to 2009 also contributed to difficulties in making the population groups comparable. Weighting as described in the methods section was used to adjust for these sampling differences. Mobile telephones were not included in the sample frames prior to 2009. Any bias from this source should be minimal as in 2004, the time of the previous survey, Australia still relied predominantly on land lines. The data is self-reported and therefore may be vulnerable to social desirability bias.

Further research is needed in translating these results into policy and practice. The findings of this research identify knowledge gaps in the length of time a baby should be breastfed and the benefits of breastfeeding for the mother and baby. It is likely that including specific information about the benefits of breastfeeding for mother and babies in community wide education campaigns would be beneficial. Differences between men’s and women’s perceptions of breastfeeding benefits, barriers and enablers need to be investigated further so that ways that men can more effectively understand and support breastfeeding mothers are identified.

## Conclusions

Being female, rating breastfeeding as important, having a belief that babies should be breastfed at least for some time and education accounted for most of the statistically significant associations in breastfeeding perceptions. Knowledge of the specific benefits of breastfeeding is relatively low. The barriers that people report are not related to any socio demographic variables so there is a high degree of uniformity about the perception of barriers to breastfeeding within the community. A number of enabling factors were identified and these should be taken into consideration when planning interventions to increase the knowledge regarding breastfeeding and the length of time that Australian women should be encouraged to breastfeed. The differences between male and female perceptions require investigation particularly in relation to returning to work.
